# Tobacco use, self-reported professional dental cleaning habits, and lung adenocarcinoma diagnosis are associated with bronchial and lung microbiome alpha diversity

**DOI:** 10.1186/s12931-024-02750-0

**Published:** 2024-03-18

**Authors:** Alexa A. Pragman, Shane W. Hodgson, Tianhua Wu, Allison Zank, Rosemary F. Kelly, Cavan S. Reilly, Chris H. Wendt

**Affiliations:** 1 Department of Medicine, Minneapolis VA Health Care System and University of Minnesota, 111F, 1 Veterans Dr, Minneapolis, MN 55417 USA; 2 Research Service, Minneapolis VA Health Care System, Minneapolis, MN USA; 3 Division of Biostatistics, University of Minnesota, Minneapolis, MN USA; 4 Department of Surgery, Minneapolis VA Health Care System and University of Minnesota, Minneapolis, MN USA

**Keywords:** Tobacco, *Haemophilus*, Lung, Microbiota, Adenocarcinoma

## Abstract

**Rationale:**

The lung microbiome is an inflammatory stimulus whose role in the development of lung malignancies is incompletely understood. We hypothesized that the lung microbiome associates with multiple clinical factors, including the presence of a lung malignancy.

**Objectives:**

To assess associations between the upper and lower airway microbiome and multiple clinical factors including lung malignancy.

**Methods:**

We conducted a prospective cohort study of upper and lower airway microbiome samples from 44 subjects undergoing lung lobectomy for suspected or confirmed lung cancer. Subjects provided oral (2), induced sputum, nasopharyngeal, bronchial, and lung tissue (3) samples. Pathologic diagnosis, age, tobacco use, dental care history, lung function, and inhaled corticosteroid use were associated with upper and lower airway microbiome findings.

**Measurements and Main Results:**

Older age was associated with greater Simpson diversity in the oral and nasopharyngeal sites (*p* = 0.022 and *p* = 0.019, respectively). Current tobacco use was associated with greater lung and bronchus Simpson diversity (*p* < 0.0001). Self-reported last profession dental cleaning more than 6 months prior (vs. 6 or fewer months prior) was associated with lower lung and bronchus Simpson diversity (*p* < 0.0001). Diagnosis of a lung adenocarcinoma (vs. other pathologic findings) was associated with lower bronchus and lung Simpson diversity (*p* = 0.024). Last professional dental cleaning, dichotomized as ≤ 6 months vs. >6 months prior, was associated with clustering among lung samples (*p* = 0.027, R^2^ = 0.016). Current tobacco use was associated with greater abundance of pulmonary pathogens *Mycoplasmoides* and *Haemophilus* in lower airway samples. Self-reported professional dental cleaning ≤ 6 months prior (vs. >6 months prior) was associated with greater bronchial *Actinomyces* and lung *Streptococcus* abundance. Lung adenocarcinoma (vs. no lung adenocarcinoma) was associated with lower *Lawsonella* abundance in lung samples. Inhaled corticosteroid use was associated with greater abundance of *Haemophilus* among oral samples and greater *Staphylococcus* among lung samples.

**Conclusions:**

Current tobacco use, recent dental cleaning, and a diagnosis of adenocarcinoma are associated with lung and bronchial microbiome α-diversity, composition (β-diversity), and the abundance of several respiratory pathogens. These findings suggest that modifiable habits (tobacco use and dental care) may influence the lower airway microbiome. Larger controlled studies to investigate these potential associations are warranted.

**Supplementary Information:**

The online version contains supplementary material available at 10.1186/s12931-024-02750-0.

## Introduction

Historically, the healthy lung was thought to be free of bacteria. The advent of next-generation sequencing techniques enabled the detailed description of lung-resident bacteria, which enter the lung by microaspiration, mucosal dispersion, inhalation, or hematogenous spread. Lung-resident bacteria may expand their population via reproduction, or be removed by ciliary action, expectoration, or the immune system. Both the adapted island model of biogeography and the neutral theory of community ecology have been used to model the relationship between the lung microbiome and its predominant source, the oral microbiome [1–4]. Regardless of the relative merits of each model, lung-resident bacteria may provoke an inflammatory response that influences lung health acutely (e.g., bacterial pneumonia, acute exacerbation of COPD) and chronically (e.g., progression of COPD [5–8]). Due to the similarities between the lung microbiome and its source (the oral microbiome), as well as the much lower biomass of the lung microbiome in comparison to the oral microbiome, great care must be taken to avoid upper airway contamination of lung microbiome samples during sampling procedures.

The lung microbiome is a key correlate of chronic inflammatory lung disorders, whose characteristics may modulate the local immune tone and affect disease outcomes [9–12]. The lung microbiome of inflammatory disorders such as chronic obstructive pulmonary disease (COPD) has received considerable study over recent years; however we know less about the lung microbiome in lung malignancy [13]. Select microbes have been implicated in lung cancer development (e.g., *M. tuberculosis* [14]), but there is little consensus on how the lung microbiome—the entire community of organisms found in the lung or lower airways—may influence lung cancer development [15–22]. Many of the microbiome characteristics that correlate with lung malignancy are also correlates of co-morbid lung diseases such as COPD—raising concern that any changes identified in these studies are not the result of lung malignancy alone.

We hypothesized that the lung microbiome correlates with multiple clinical factors, including the presence of a lung malignancy. We undertook the present prospective cohort study of surgically obtained lung tissue samples from subjects undergoing lung lobectomy for suspected or confirmed lung cancer to accomplish two goals: evaluate for malignancy-associated microbiome features and evaluate for other clinical factors (i.e., COPD, tobacco use, oral care habits) associated with lung microbiome features. Our surgically obtained low biomass lung samples were not passed through a bronchoscope or the high biomass upper airway. This minimized potential contamination of our lung samples by DNA from the bronchoscope or the upper airway and permitted detailed analyses of correlations between multiple clinical factors and the characteristics of the lung microbiome.

## Methods

### Study design and recruitment

We conducted a prospective observational study of patients undergoing lung lobectomy at the Minneapolis VA Medical Center (MVAMC) for suspected or confirmed lung cancer. To decrease the influence of medications on our findings, we excluded subjects who had used antibiotics or systemic corticosteroids in the prior 1 month. The protocol was approved by the Minneapolis VA IRB (#4348-B) and in accordance of the Declaration of Helsinki. Additional details on the methods employed are provided in the supplementary information. Some of these findings have been presented previously in the form of an abstract [23].

### Sample collection procedures

The day before surgery, we collected the first oral wash sample and an induced sputum sample. The morning of surgery, we collected the second oral wash sample. In the operating room we collected samples from the resected lobe, taking care to avoid the lung tumor and sample the adjacent healthy-appearing lung tissue. Three separate samples were obtained from the lung parenchyma by cutting open the distal lung tissue and vigorously swabbing the alveolar air spaces for 30 s. Bronchial samples were obtained from the main bronchial airway supplying the removed lobe using a nylon-flocked swab (Copan Diagnostics, Murrieta, CA). Nasopharyngeal samples were obtained by swabbing the nasopharynx for 15 s. Negative control samples consisted of unused sterile water or unused swabs which were processed concurrently with subject samples.

### Sample processing, 16 S rRNA gene quantification, and MiSeq sequencing and processing

All samples and negative controls were extracted using the MO BIO PowerSoil DNA Isolation Kit (QIAGEN, Germantown, MD). Extracted DNA from each sample was submitted to the University of Minnesota Genomics Center for 16 S rRNA gene quantification using droplet digital PCR (ddPCR) and 16 S rRNA gene V4 MiSeq sequencing. 16 S rRNA V4 sequences were processed and analyzed as described in the supplementary information. Additional information on subject samples, negative controls, 16 S rRNA V4 sequencing and processing is available in the Supplementary Materials. Contaminant taxa were removed from the dataset as described in the supplementary information before further analysis (Figure S1 and S2). Data are publicly available at NCBI SRA (PRJNA1006673).

### Statistical analyses

All analyses were conducted in R version 3.6.0. Linear regression (LR) was used for analyses with independent data points. Analyses incorporating repeated measures from the same site or subject employed generalized estimating equation (GEE) with an independence correlation structure. GEE models utilized indicators for sample type followed by a post-hoc analyses to compare all levels of sample type, followed by implementation of adjusted *p* values based on the joint normal or t distribution of the linear function. PERMANOVA analyses of β-diversity were performed for each site individually. Taxonomic associations were determined independently for each combination of the 6 clinical characteristics and 5 sample sites. For each sample site (oral wash, nasopharynx, sputum, lung, and bronchus) only genera present in at least 10% of samples at that site were included (oral wash 70 genera, sputum 74 genera, nasopharynx 50 genera, bronchus 29 genera, and lung 20 genera). *P*-values were adjusted using the Holm method.

## Results

### Study subjects

Among subjects undergoing lung lobectomy for suspected or confirmed lung cancer at the Minneapolis VA Medical Center (MVAMC) and who reported no use of systemic antibiotics or systemic corticosteroids in the prior 1 month, 44 consented to study participation (Table 1). Consistent with the VA patient population, most subjects were male. Thirty-six of 44 (81.8%) met spirometric criteria for COPD, and over half (20, 55.6%) of those with COPD had mild obstruction. Few of those with COPD (2, 5.6%) were using inhaled corticosteroids (ICS). Subjects with COPD (vs. without COPD) reported a greater number of pack-years of tobacco exposure (49.5 vs. 25) and were more likely to report current alcohol use (66.7% vs. 25%). Most subjects were found to have lung adenocarcinoma (26, 59.1%), although 2 subjects had more than one lung malignancy and 3 subjects did not have a malignancy. Additionally, in 2 subjects a pathologic diagnosis was not available.

**Table 1:** Subject baseline characteristics

	Non-COPD (*N* = 8)	COPD (*N* = 36)	Overall (*N* = 44)	*p* value^*^
Gender, Male (%)	8 (100)	34 (94.4)	42 (95.5)	1.00
Age, median (IQR)	68.5 (6)	67 (9)	67 (9)	0.953
Race, Caucasian white (%)	7 (87.5)	34 (94.4)	41 (93.2)	0.461
Diabetes, Yes (%)	1 (12.5)	3 (8.3)	4 (9.1)	0.566
Gastroesophageal reflux disease, Yes (%)	4 (50)	17 (47.2)	21 (47.7)	1.00
COPD Severity (%)				
Mild	0 (0)	20 (55.6)		
Moderate	0 (0)	14 (38.9)		
Severe	0 (0)	2 (5.6)		
FEV_1_% predicted, median (IQR)	91.65 (25.45)	80.25 (20.75)	82 (20.25)	0.038
Inhaled corticosteroids, Yes (%)	0 (0)	2 (5.6)	2 (4.5)	1.00
Pack-years of smoking, median (IQR)^a^	25 (35.5)	49.5 (21.25)	46.5 (30)	0.018
Current tobacco use, Yes (%)	1 (12.5)	4 (11.1)	5 (11.4)	1.00
Current alcohol use, Yes (%)	2 (25)	24 (66.7)	26 (59.1)	0.048
Last dental visit *≤* to 6 months (%)^b^	3 (42.9)	12 (44.4)	15 (44.1)	1.00
Oral steroid or antibiotic use in last 2 months, Yes (%)	0 (0)	0 (0)	0 (0)	
Adenocarcinoma, Yes (%)^c^	5 (62.5)	21 (58.3)	26 (59.1)	1.00
Squamous cell carcinoma, Yes (%)^c^	1 (12.5)	9 (25)	10 (22.7)	0.659
Large cell neuroendocrine tumor, Yes (%)	0 (0)	2 (5.6)	2 (4.5)	1.00
Metastatic colorectal adenocarcinoma, Yes (%)	1 (12.5)	0 (0)	1 (2.3)	0.182
Unidentified carcinoma, Yes (%)	0 (0)	1 (2.8)	1 (2.3)	1.00
No evidence of malignancy, Yes (%)	1 (12.5)	2 (5.6)	3 (6.8)	0.461
No pathologic diagnosis, Yes (%)	0 (0)	2 (5.6)	2 (4.5)	1.00

Frequencies and percentages are presented unless specified otherwise. COPD, chronic obstructive pulmonary disease; FEV1% predicted, forced expiratory volume in 1 s, percent of predicted value; IQR, interquartile range.

^*^A Two-Sample t test was conducted for all continuous variables and a Fisher exact test for all categorical variables.

^a^One subject was a never-smoker, all others reported current or former tobacco use.

^b^Ten subjects did not provide dental visit information because they were edentulous

^c^One subject had 2 independent adenocarcinomas. One subject had 3 tumors (2 adenocarcinomas and 1 squamous cell carcinoma)

### Microbiome biomass

**Fig. 1:**
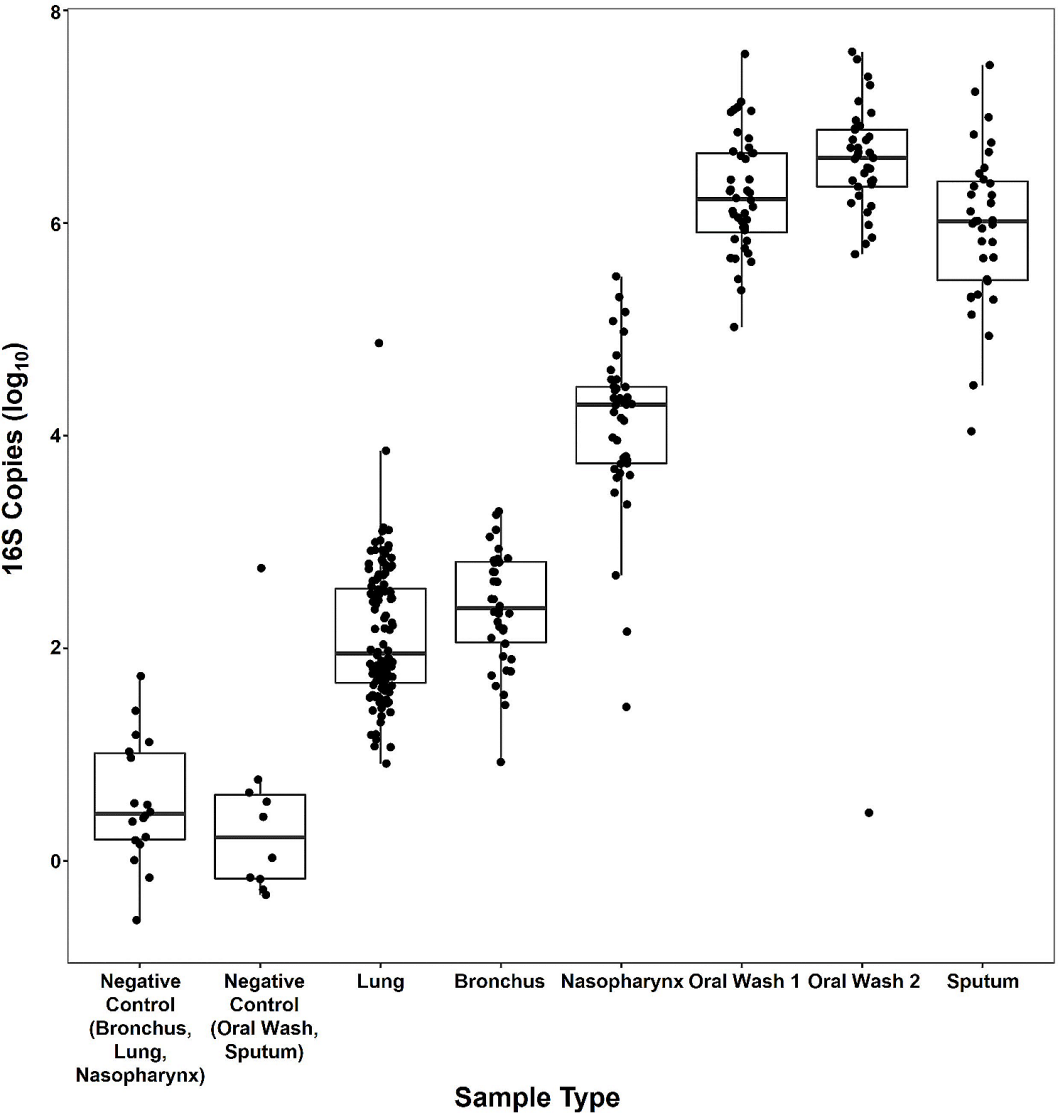
Upper and lower airway biomass. Horizontal lines represent the median value in each group, while the top and bottom of the boxes represent the 75th and 25th percentile values, respectively. Both negative control sample types had significantly lower biomass than all patient sample types (GEE, all *p* < 0.001). The lowest biomass samples (lung, bronchus) had lower biomass than all other subject sample types (GEE, all *p* < 0.001). The highest biomass samples (oral wash, sputum) had higher biomass than all other subject samples (GEE, all *p* < 0.001). Lung and bronchus samples were not significantly different from each other, and oral washes and sputum samples were not significantly different from each other

All subject samples and negative controls underwent biomass quantification with droplet digital PCR (ddPCR) prior to microbiome sequencing. Both negative control sample types had significantly less biomass than all subject sample types (generalized estimating equations [GEE] model with single-step adjustment, *p* < 0.001; Table S1, Fig. 1). Lung and bronchus samples had lower biomass than nasopharyngeal, oral wash, and sputum samples (GEE, all *p* < 0.001). Oral wash and sputum samples had higher biomass than lung, bronchus, and nasopharyngeal samples (GEE, all *p* < 0.001). Nasopharyngeal sample biomass was significantly different from the other sample types (GEE, *p* < 0.001).

### Alpha diversity

**Fig. 2:**
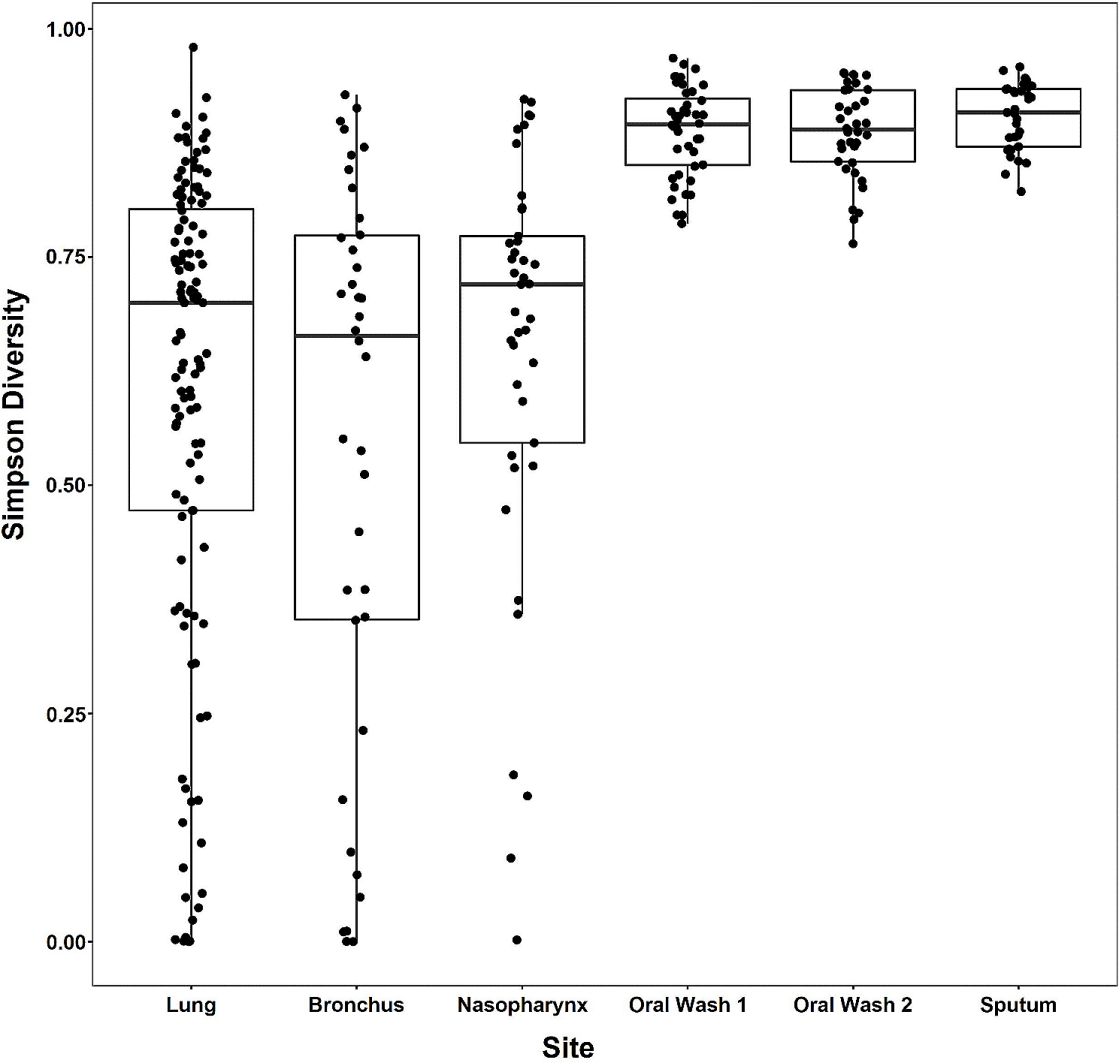
Oral wash and sputum samples have greater Simpson diversity than lung, bronchus, and nasopharyngeal samples. Simpson diversity was determined for each sample and illustrated by sample site. Horizontal bars represent the median value for each sample while the top and bottom of the boxes represent the 75th and 25th percentile values, respectively. Oral wash and sputum samples are significantly more diverse than lung, bronchus, and nasopharyngeal samples (GEE, *p* < 0.0001)

α-diversity statistics (Simpson diversity, Shannon diversity, and Chao1 diversity) were calculated for all samples and illustrated by sample type. Simpson diversity findings were generally consistent across the other α-diversity metrics and are presented here. Oral wash and sputum samples are significantly more diverse than bronchus and lung samples (GEE, *p* < 0.0001; Fig. 2). Bronchus, lung, and nasopharyngeal sample Simpson diversity are not significantly different from each other. Shannon diversity findings are similar to Simpson diversity findings, however Chao1 diversity findings demonstrate that nasopharyngeal samples are similar in richness to oral wash and sputum samples, which all have greater richness than bronchus and lung samples (Figure S3). Bronchus Simpson diversity associates with within-subject oral wash and sputum Simpson diversity, but not within-subject lung or nasopharyngeal diversity (GEE, Table S2).

**Fig. 3:**
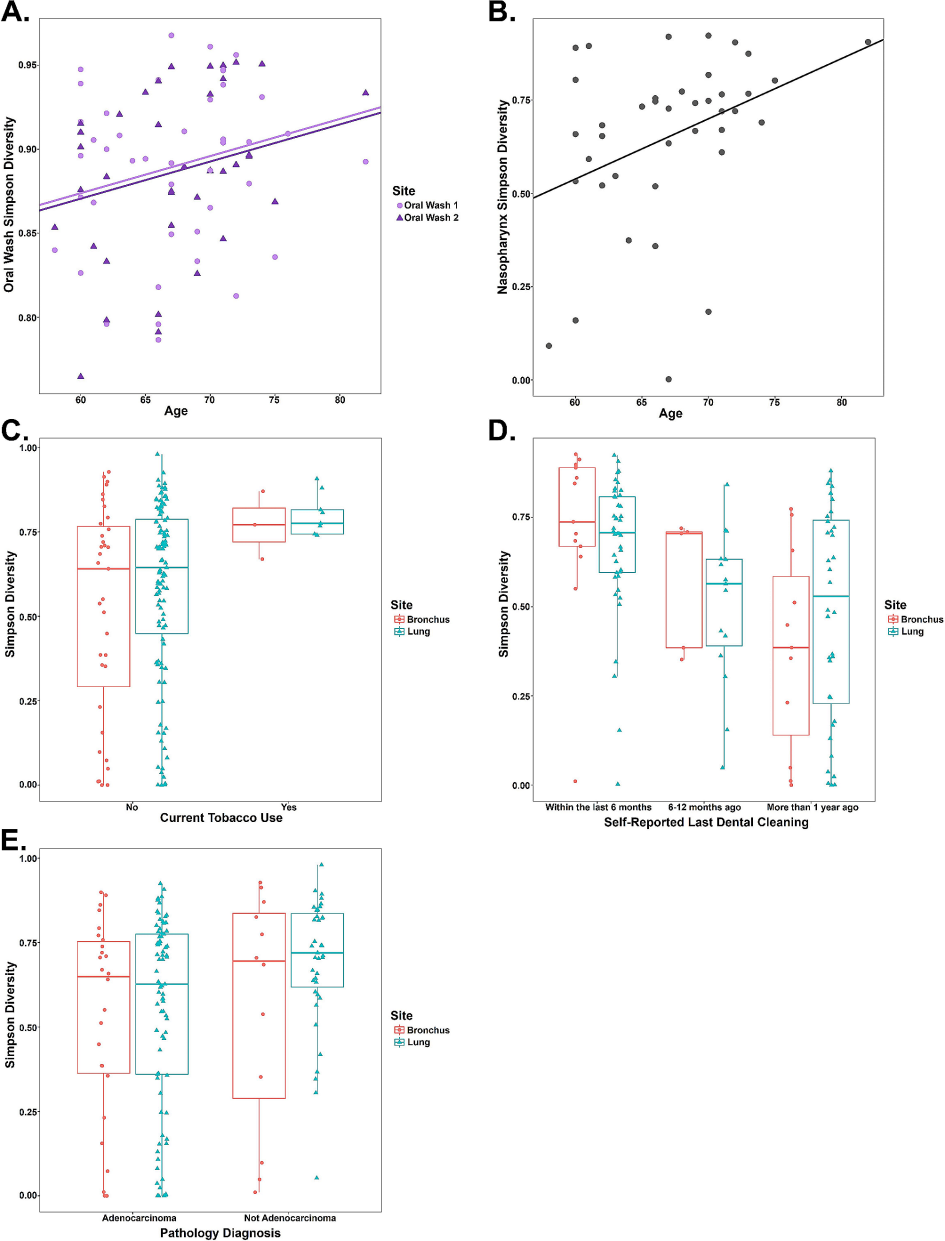
Clinical characteristics are associated with upper and lower airway Simpson diversity. **A.**. Older age is associated with greater oral wash Simpson diversity. Oral wash 1 and oral wash 2 are illustrated by shape, and the regression lines represent the association between older age and greater Simpson diversity (GEE, CE 0.0022, 95% CI [0.00024, 0.0042], *p* = 0.022). **B**. Older age is associated with greater nasopharyngeal Simpson diversity. The regression line represents the association between older age and greater Simpson diversity (LR, CE 0.016, 95% CI [0.0032, 0.029], *p* = 0.019). **C.** Current tobacco use is associated with greater lung and bronchus Simpson diversity (GEE, CE 0.22, 95% CI [0.17, 0.28], *p* < 0.0001). **D.** Last professional dental cleaning more than 6 months prior to surgery (vs. within the last 6 months) was associated with lower lung and bronchus Simpson diversity (GEE, CE 0.21, 95% CI [0.12, 0.29], *p* < 0.0001). Edentulous subjects were excluded from this analysis. Data were dichotomized at the 6 month timepoint for the GEE analysis, however three timepoints (within the last 6 months, 6–12 months ago, and more than 1 year ago) are presented here for illustrative purposes. **E**. Lung adenocarcinoma (vs. other pathologic finding) was associated with lower lung and bronchus Simpson diversity (GEE, CE -0.11, 95% CI [-0.21, -0.015], *p* = 0.024)

We also investigated whether 6 relevant clinical characteristics were associated with α-diversity at any of the 5 anatomic sites. Older age was associated with greater Simpson diversity in the oral and nasopharyngeal sites (GEE, Coefficient Estimate [CE] 0.0022, 95% Confidence Interval [CI, 0.00024, 0.0042], *p* = 0.022; and LR, CE 0.016, 95% CI [0.0032, 0.029], *p* = 0.019, respectively; Fig. 3a and b). Current tobacco use was associated with greater lung and bronchus Simpson diversity (GEE, CE 0.22, 95% CI [0.17, 0.28], *p* < 0.0001, Fig. 3c), but not diversity at other sites.

Dental care habits were also associated with Simpson diversity in lower airway samples. Ten of the 44 subjects were edentulous and therefore not included in this analysis (Figure S4). Among the 34 remaining subjects, 15 reported that their last professional dental cleaning was within the prior 6 months, while 19 reported that their last professional dental cleaning was more than 6 months prior. Self-reported last profession dental cleaning more than 6 months prior was associated with lower lung and bronchus Simpson diversity (GEE, CE 0.21, 95% CI [0.12, 0.29], *p* < 0.0001, Fig. 3d). A pathologic diagnosis of lung adenocarcinoma was made in 26 (59.1%) of subjects, while the remaining subjects were diagnosed with other malignancies (13, 29.5%) or non-malignant findings (5, 11.4%). Presence of a lung adenocarcinoma (vs. other pathologic findings) was associated with lower bronchus and lung Simpson diversity (GEE, CE -0.11, 95% CI [-0.21, -0.015], *p* = 0.024, Fig. 3e). Unless mentioned above, tests of association between site-specific Simpson diversity and these 6 clinical characteristics (including FEV1pp, ICS use, and pack-years of tobacco use) were not significant.

### Beta diversity

**Fig. 4:**
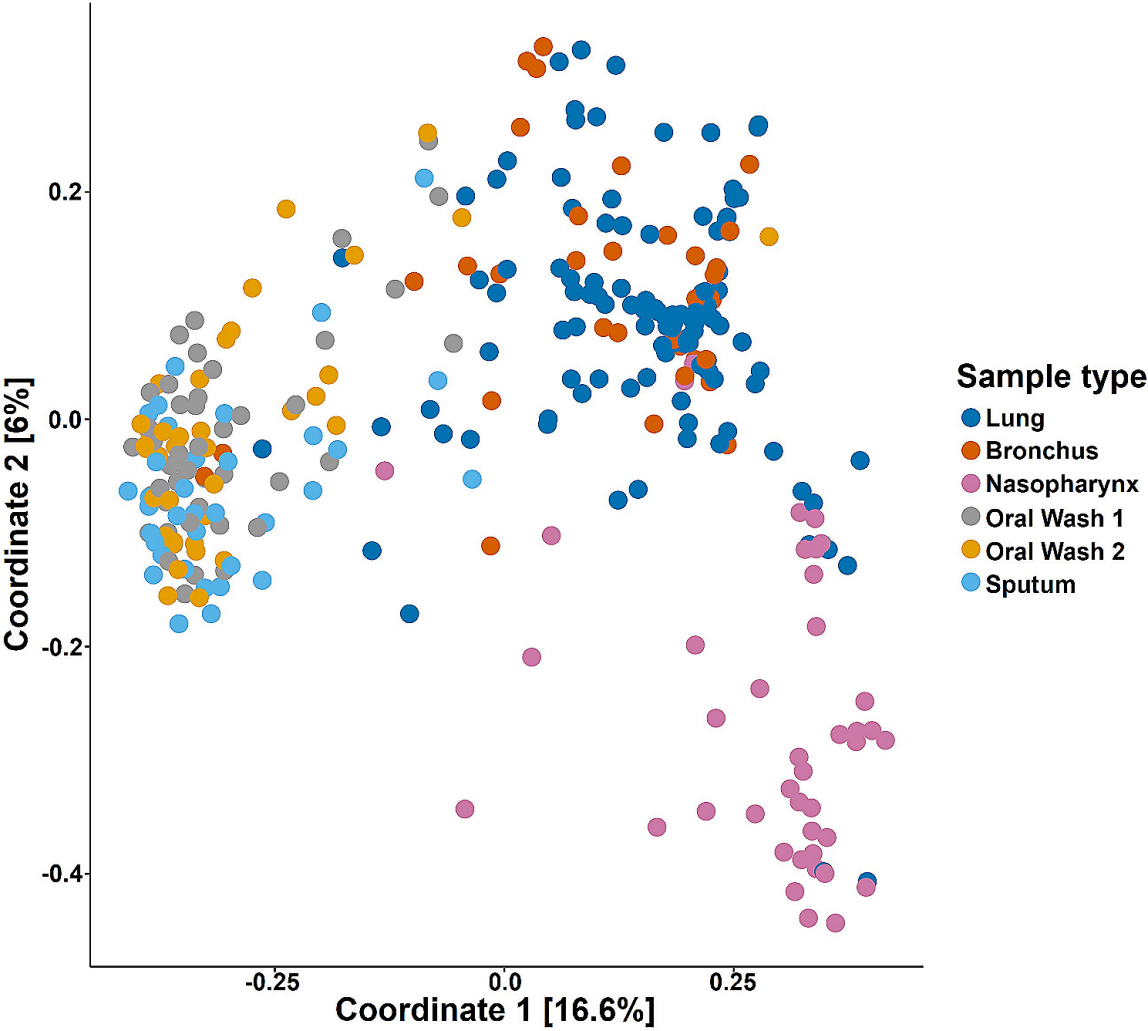
Principal coordinate analysis (PCoA) illustrates sample clustering by sampling site. All subject samples were assessed by Bray-Curtis dissimilarity and illustrated by PCoA of Coordinate 1 (16.6% of variance) and Coordinate 2 (6% of variance). Lung and bronchus samples are similar to each other, and cluster separately from the nasopharyngeal samples and the oral wash and sputum samples. Oral wash and sputum samples cluster with each other, separate from the other samples

β-diversity was assessed using Bray-Curtis dissimilarity and illustrated with principal coordinates analysis (PCoA; Fig. 4). Coordinate 1, representing 16.6% of the variance within the dataset, separates nasopharyngeal, lung and bronchus samples from oral wash and sputum samples. Coordinate 2, representing 6% of the variance, separates nasopharyngeal samples from the other sample types. β-diversity was also illustrated using weighted UniFrac, with similar separation by anatomic site (Figure S5).

#### PERMANOVA analyses

**Fig. 5:**
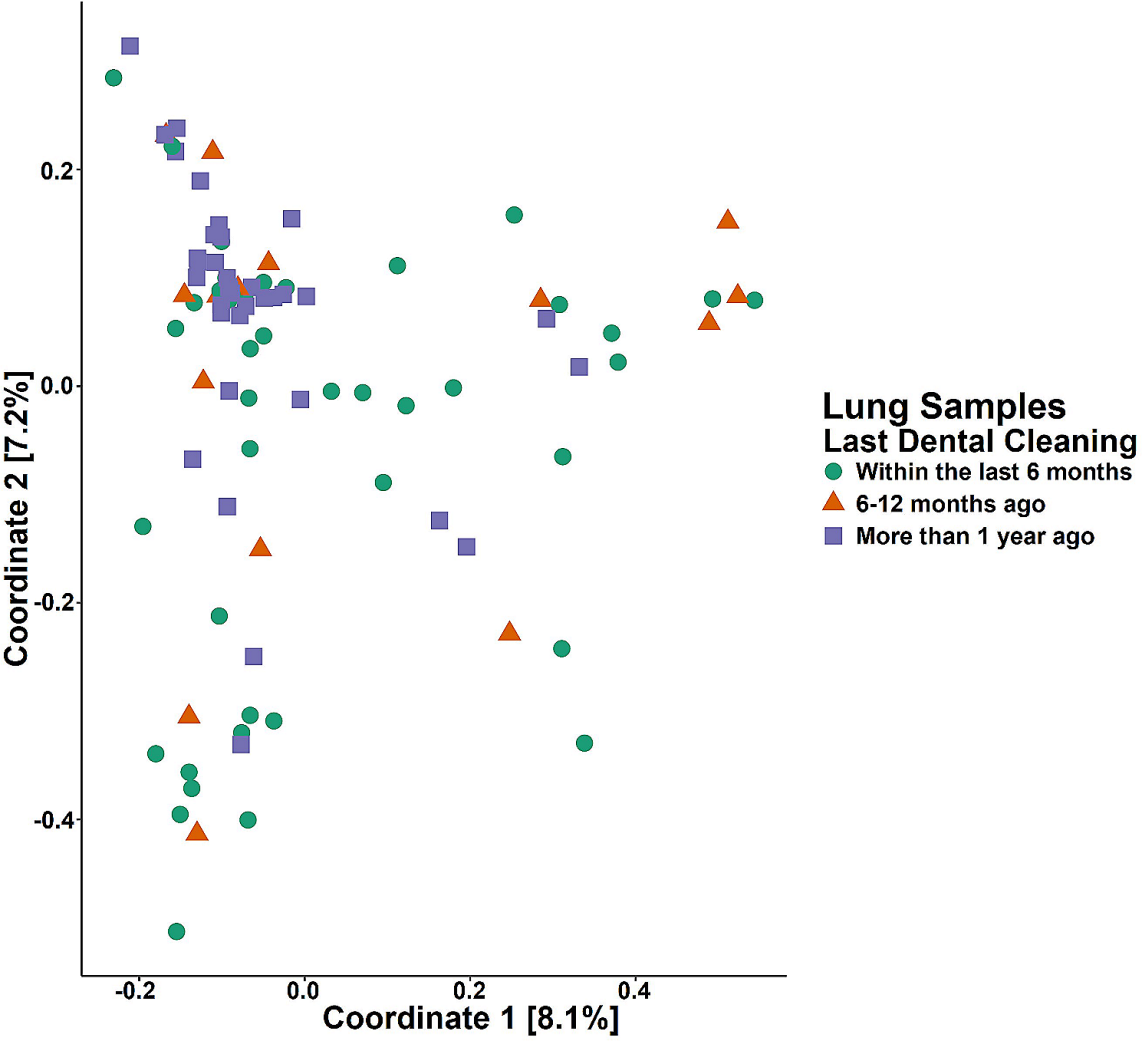
Principal coordinate analysis (PCoA) illustrates lung sample clustering by self-reported last professional dental cleaning. All lung samples were assessed by Bray-Curtis dissimilarity and illustrated by PCoA of Coordinate 1 (8.1% of variance) and Coordinate 2 (7.2% of variance). Samples were dichotomized by self-reported last dental cleaning (within the last 6 months vs. more than 6 months ago) for analysis, although samples are labeled here with additional detail. Self-reported last professional dental cleaning was associated with lung microbiome composition (*p* = 0.027, R^2^ = 0.016)

After separating samples based on site, correlations between clinical characteristics and β-diversity were assessed with PERMANOVA analyses utilizing Bray-Curtis dissimilarity. Last professional dental cleaning, dichotomized as within the last 6 months vs. more than 6 months prior, was associated with clustering among lung samples (*p* = 0.027, R^2^ = 0.016; Fig. 5). Several other clinical characteristics resulted in *p*-values between 0.05 and 0.10, which did not reach statistical significance (Figure S6). FEV1pp, ICS use, and current tobacco use were not associated with microbiome composition in our dataset. PERMANOVA analyses utilizing the weighted UniFrac matrix yielded very similar results (Table S3).

### Bacterial taxa

We investigated correlations between relevant clinical factors and taxonomic composition. Increasing age was associated with greater abundance of 3 oral taxa (Table 2). Current tobacco use was associated with changes in abundance of multiple oral and lung taxa, including greater abundance of pulmonary pathogens *Mycoplasmoides* and *Haemophilus* in lower airway samples (Table 3). Self-reported recent professional dental cleaning (within the last 6 months) was associated with greater bronchial *Actinomyces* and lung *Streptococcus* abundance, compared with less recent professional dental cleaning (more than 6 months ago; Table 4). A diagnosis of lung adenocarcinoma (vs. no diagnosis of lung adenocarcinoma) was associated with lower *Lawsonella* abundance in lung samples (Table 5). The use of ICS was associated with changes in taxa abundance in the oral, lung, nasopharyngeal, and sputum microbiome. Notably, these included greater abundance of *Haemophilus* among oral samples, greater *Staphylococcus* among lung samples, and lower *Fusobacterium, Gemella*, and *Acinetobacter* among lung samples (Table 6). Finally, greater FEV1pp was associated with lower *Escherichia/Shigella* abundance in the lung microbiome (Table 7).

**Table 2:** Taxa whose frequency was associated with age

Anatomic Site	Genera	Coefficient	Adjusted *p* value
Oral Wash	*Megasphaera*	0.1571	0.003
Oral Wash	*Lautropia*	0.1580	0.002
Oral Wash	*Neisseria*	0.2290	0.001

**Table 3:** Taxa whose frequency was associated with current tobacco use

Anatomic Site	Genera	Coefficient	Adjusted *p* value
Oral Wash	*Bifidobacterium*	-1.1686	< 0.0001
Oral Wash	*Metaprevotella*	-0.5907	0.048
Oral Wash	*Zea*	-0.4620	0.024
Oral Wash	*Butyrivibrio*	-0.3385	0.047
Oral Wash	*Amniculibacterium*	0.8398	0.025
Bronchus	*Mycoplasmoides*	2.6882	0.021
Lung	*Granulicatella*	-0.7183	0.01
Lung	*Haemophilus*	1.6866	< 0.0001
Lung	*Prevotella*	2.0149	0.03

**Table 4:** Taxa whose frequency was associated with self-reported professional dental cleaning (≤ 6 months vs. >6 months)

Anatomic Site	Genera	Coefficient	Adjusted *p* value
Bronchus	*Actinomyces*	2.5723	0.028
Lung	*Streptococcus*	2.4435	0.007

**Table 5:** Taxa whose frequency was associated with adenocarcinoma

Anatomic Site	Genera	Coefficient	Adjusted *p* value
Lung	*Lawsonella*	-2.2712	0.009

**Table 6:** Taxa whose frequency was associated with ICS use

Anatomic Site	Genera	Coefficient	Adjusted *p* value
Oral Wash	*Neisseria*	-2.3569	< 0.0001
Oral Wash	*Amniculibacterium*	-2.3309	< 0.0001
Oral Wash	*Centipeda*	-2.2185	< 0.0001
Oral Wash	*Oribacterium*	-2.1467	0.016
Oral Wash	*Mogibacterium*	-1.6824	< 0.0001
Oral Wash	*Lautropia*	-1.4135	< 0.0001
Oral Wash	*Campylobacter*	-1.4025	< 0.0001
Oral Wash	*Actinobacillus*	-1.2966	0.035
Oral Wash	*Catonella*	-1.1469	< 0.0001
Oral Wash	*Moryella*	-0.9571	0.0009
Oral Wash	*Cardiobacterium*	-0.8282	< 0.0001
Oral Wash	*Schwartzia*	-0.7803	< 0.0001
Oral Wash	*Peptostreptococcus*	-0.7727	0.0002
Oral Wash	*Alloscardovia*	-0.7287	0.0006
Oral Wash	*Filifactor*	-0.6678	0.008
Oral Wash	*Solobacterium*	-0.6282	< 0.0001
Oral Wash	*Abiotrophia*	-0.6060	0.006
Oral Wash	*Zea*	-0.4380	0.019
Oral Wash	*Butyrivibrio*	-0.3209	0.037
Oral Wash	*Haemophilus*	1.7478	< 0.0001
Lung	*Fusobacterium*	-0.8819	0.0006
Lung	*Gemella*	-0.5366	0.012
Lung	*Acinetobacter*	-0.4807	0.022
Lung	*Staphylococcus*	2.3731	< 0.0001
Nasopharyngeal	*Corynebacterium*	-3.9565	0.003
Sputum	*Phocaeicola*	4.4411	< 0.0001

**Table 7:** Taxa whose frequency was associated with greater FEV1pp.

Anatomic Site	Genera	Coefficient	Adjusted *p* value
Lung	*Escherichia.Shigella*	-0.0464	0.018

## Discussion

Our prospective observational study of the upper and lower airway microbiome of subjects undergoing lung lobectomy for suspected or confirmed lung cancer identified tobacco use, dental care habits, and lung histopathology as clinical correlates of the lower airway microbiome. Utilizing lung lobectomy specimens to study the lower airway microbiome minimized potential upper airway contamination of our low biomass lung and bronchial samples. This, coupled with our use of touchdown PCR and inclusion of multiple negative control samples, allowed us to examine clinical correlates of the lower airway microbiome.

In contrast to several of our previous studies [8, 24, 25], we did not detect many clinical correlates of the upper airway microbiome (oral, nasopharyngeal, or sputum), perhaps because the current study population had milder obstruction compared with prior study populations (current study median FEV1pp 82% vs. prior work median FEV1pp 48% [8]). This study identified age, tobacco use, and ICS use as being associated with α-diversity (age alone) and taxonomic abundance (all 3 factors) of the oral—but not sputum—microbiome. Certainly, tobacco and ICS can be deposited onto the oral mucosa and may directly influence the oral microbiome. However, the finding that increasing age is associated with greater oral microbiome α-diversity is unexpected. Our previous studies and the literature revealed a relationship between increasing age and *lower* sputum α-diversity [8, 26, 27]. There are several explanations for this potential discrepancy. The presence of a malignancy or the milder airway obstruction may have influenced our age-related findings. Previous studies of the sputum microbiome recruited subjects of similar age to our present subjects (mean or median age in the late 60’s), however the other studies recruited patients with more severe COPD. Subjects in this study had a median FEV1pp of 82% (IQR 20%), few subjects using ICS (2, 4.5%), and very few subjects who meet criteria for the “frequent exacerbator” phenotype. In contrast, other studies addressing correlations between age and the upper airway microbiome studied subjects with more severe airflow obstruction (FEV1pp median 48%, IQR 19%), more use of ICS, and specifically recruited the subjects with the more severe “frequent exacerbator” phenotype. It is possible that age is a marker of increased exposure to antibiotics among those at high risk of COPD exacerbations, and cumulative antibiotic use is responsible for lowering α-diversity. Additionally, the presence of a malignancy may influence our age-related findings. Alternatively, the associations between age and α-diversity noted here and in the above-mentioned studies may differ based on other markers of COPD severity (e.g., FEV1pp). Additional studies powered to simultaneously model age, FEV1pp, exacerbation phenotype, and historic antibiotic use may be needed to further address this issue.

We found that current tobacco use among a small number of subjects was associated with lower airway microbiome features, namely greater Simpson diversity and greater abundance of *Mycoplasma* and *Haemophilus* (two well-known respiratory pathogens) in the lung and bronchial samples. Several prior studies of the lung microbiome of current tobacco users vs. non-users did not identify changes in α-diversity or taxonomic composition that correlated with tobacco use [3, 28]. However, one recent study identified a dose-dependent correlation between tobacco exposure and relative abundance of several members of the phylum Proteobacteria (which includes *Haemophilus*) in the lung microbiome [29]. Unlike prior studies, the majority of our subjects had a malignancy, which may have influenced our findings. In a mouse model of tobacco-associated lung adenocarcinoma, lung microbiome α-diversity did not change following exposure to tobacco. However, lung microbiome α-diversity increased following tobacco exposure among mice unable to express the bacterial growth inhibitor lipocalin 2, a component of the innate immune system that is protective against lung adenocarcinoma. Further studies are needed to determine if increased α-diversity has a mechanistic role in the development of lung adenocarcinoma.

Self-reported recent professional dental cleaning was also associated with multiple changes in the lung or bronchial microbiome, including greater α-diversity, shifts in community composition (β-diversity), and increases in *Actinomyces* and *Streptococcus* abundance. There is much interest in the influence that oral health and the oral microbiome have on lung health, particularly in the context of COPD [30, 31]. The presence of an oral disease may influence the type or number of bacteria aspirated from the oral cavity, the inflammatory cells or proteins aspirated from the oral cavity, or provoke systemic inflammation which subsequently worsens respiratory health [32]. While dental cleaning itself did not appreciably change the dental plaque microbiome, a recent randomized controlled trial demonstrated that professional dental cleaning every 6 months decreases COPD exacerbation frequency [31]. It is possible that professional dental cleaning modifies the lung microbiome without significant change to the oral microbiome, possibly by decreasing microbial biomass resulting in fewer inflammatory mediators that are aspirated from the oral cavity. Alternatively, in our study, recent dental cleaning may be a surrogate marker of higher income or better access to medical care [33], which may influence the microbiome.

The diagnosis of lung adenocarcinoma (vs. other malignant or non-malignant findings) was associated with lower lung and bronchial α-diversity. This finding is consistent with several human and mouse studies of the lung cancer microbiome, which show that lung adenocarcinoma is associated with lower lung microbiome diversity compared to either healthy lung tissue or squamous cell lung cancer [34–36]. Animal studies support the idea that loss of lung microbiome α-diversity and/or lung microbiome dysbiosis plays a role in lung inflammation and tumorigenesis by activating γδ T cells and IL-17 production [21, 35].

Although we did not identify associations between ICS use and α- or β-diversity, we identified more taxonomic changes associated with ICS use than any other clinical factor in our study. ICS are deposited onto the oropharynx and airways, where they decrease inflammatory cytokines and other host defense proteins. These effects lead to microbial dysbiosis (changes in β-diversity) and are likely responsible for the increased risk of pneumonia among those using ICS [37]. Consistent with prior studies [38, 39], we identified an association between ICS use and greater abundance of the respiratory pathogens *Haemophilus* and *Staphylococcus* among oral and lung samples, respectively, as well as a lesser abundance of *Fusobacteria*, *Gemella*, and *Acinetobacter* in lung samples.

Our study had several strengths as well as relative weaknesses. We were able to obtain bronchial and lung parenchymal samples from fresh lung lobectomy tissue, without passing these low biomass lung samples through the bacteria-rich upper airways. Combined with our use of multiple negative control samples, removal of potential contaminant taxa from the dataset, and touchdown PCR, we have been able to identify lower airway microbiome correlates of multiple relevant clinical factors, including lung malignancy. Additionally, our study design permitted the collection of healthy-appearing lung tissue from subjects with near-normal spirometry or mild COPD. The lung tissue microbiome of mild COPD is understudied as there are few surgical indications for lung resection among these patients. Unfortunately, we may have been underpowered for assessment of uncommon clinical factors in our dataset (ICS use, non-malignant pathologic findings). Additionally, tobacco use and dental visit history were assessed via self-report.

In conclusion, we assessed the upper and lower airway microbiome of subjects with relatively mild obstruction undergoing lung lobectomy for suspected or confirmed lung cancer. In our small study, we determined that current tobacco use, recent dental cleaning, and a diagnosis of adenocarcinoma are associated with lung and bronchial microbiome α-diversity, composition (β-diversity), and the abundance of several respiratory pathogens. Although these observational data cannot determine the cause of these microbiome findings, they are among the first findings to suggest that modifiable habits (tobacco use and dental care habits) may influence the lower airway microbiome. Larger controlled studies to investigate these potential associations are warranted.

### Electronic supplementary material

Below is the link to the electronic supplementary material.

Supplementary Material 1

## Data Availability

The datasets generated and analyzed during the current study are available in the NCBI SRA repository (PRJNA1006673), https://www.ncbi.nlm.nih.gov/sra/?term=PRJNA1006673.

## References

[1] Dickson RP, Erb-Downward JR, Huffnagle GB (2014). Towards an ecology of the lung: new conceptual models of pulmonary microbiology and pneumonia pathogenesis. Lancet Respir Med.

[2] Dickson RP, Erb-Downward JR, Freeman CM (2015). Spatial variation in the Healthy Human Lung Microbiome and the adapted Island Model of Lung Biogeography. Ann Am Thorac Soc.

[3] Morris A, Beck JM, Schloss PD (2013). Comparison of the respiratory microbiome in healthy nonsmokers and smokers. Am J Respir Crit Care Med.

[4] Pragman AA, Lyu T, Baller JA (2018). The lung tissue microbiota of mild and moderate chronic obstructive pulmonary disease. Microbiome.

[5] Sethi S, Maloney J, Grove L, Wrona C, Berenson CS (2006). Airway inflammation and bronchial bacterial colonization in chronic obstructive pulmonary disease. Am J Respir Crit Care Med.

[6] Sethi S, Murphy TF (2001). Bacterial infection in chronic obstructive pulmonary disease in 2000: a state-of-the-art review. Clin Microbiol Rev.

[7] Desai H, Eschberger K, Wrona C (2014). Bacterial colonization increases daily symptoms in patients with chronic obstructive Pulmonary Disease. Ann Am Thorac Soc.

[8] Pragman AA, Hodgson SW, Wu T, Zank A, Reilly CS, Wendt CH. Sputum microbiome α-diversity is a key feature of the COPD frequent exacerbator phenotype. European Respiratory Journal Open Research. 2023. In press. 10.1183/23120541.00595-2023 PMC10851948 38333651

[9] Dicker AJ, Huang JTJ, Lonergan M (2021). The sputum microbiome, airway inflammation, and mortality in chronic obstructive pulmonary disease. J Allergy Clin Immunol.

[10] Ghebre MA, Pang PH, Diver S (2018). Biological exacerbation clusters demonstrate asthma and chronic obstructive pulmonary disease overlap with distinct mediator and microbiome profiles. J Allergy Clin Immunol.

[11] Wang Z, Locantore N, Haldar K (2021). Inflammatory endotype-associated Airway Microbiome in Chronic Obstructive Pulmonary Disease Clinical Stability and exacerbations: a Multicohort Longitudinal Analysis. Am J Respir Crit Care Med.

[12] Segal LN, Alekseyenko AV, Clemente JC (2013). Enrichment of lung microbiome with supraglottic taxa is associated with increased pulmonary inflammation. Microbiome.

[13] Klein M, Pragman AA, Wendt C (2022). Biomarkers and the microbiome in the detection and treatment of early-stage non-small cell lung cancer. Semin Oncol.

[14] Abdeahad H, Salehi M, Yaghoubi A, Aalami AH, Aalami F, Soleimanpour S (2022). Previous pulmonary tuberculosis enhances the risk of lung cancer: systematic reviews and meta-analysis. Infect Dis (Lond).

[15] Greathouse KL, White JR, Vargas AJ (2018). Interaction between the microbiome and TP53 in human lung cancer. Genome Biol.

[16] Huang D, Su X, Yuan M (2019). The characterization of lung microbiome in lung cancer patients with different clinicopathology. Am J Cancer Res.

[17] Jin J, Gan Y, Liu H (2019). Diminishing microbiome richness and distinction in the lower respiratory tract of lung cancer patients: a multiple comparative study design with independent validation. Lung Cancer.

[18] Kovaleva O, Podlesnaya P, Rashidova M (2020). Lung Microbiome differentially impacts Survival of patients with Non-small Cell Lung Cancer depending on Tumor Stroma phenotype. Biomedicines.

[19] Nejman D, Livyatan I, Fuks G (2020). The human tumor microbiome is composed of tumor type-specific intracellular bacteria. Science.

[20] Patnaik SK, Cortes EG, Kannisto ED (2021). Lower airway bacterial microbiome may influence recurrence after resection of early-stage non-small cell lung cancer. J Thorac Cardiovasc Surg.

[21] Tsay JJ, Wu BG, Sulaiman I (2021). Lower Airway Dysbiosis affects Lung Cancer Progression. Cancer Discov.

[22] Tsay J-CJ, Wu BG, Badri MH (2018). Airway Microbiota is Associated with Upregulation of the PI3K pathway in Lung Cancer. Am J Respir Crit Care Med.

[23] editors. Lung Microbiome Correlates With Lung Adenocarcinoma and Self-reported Dental Cleaning. 2023; Washington, DC: 2023.

[24] Pragman AA, Knutson KA, Gould TJ, Isaacson RE, Reilly CS, Wendt CH (2019). Chronic obstructive pulmonary disease upper airway microbiota alpha diversity is associated with exacerbation phenotype: a case-control observational study. Respir Res.

[25] Pragman AA, Knutson KA, Gould TJ (2019). Chronic obstructive pulmonary disease upper airway microbiome is associated with select clinical characteristics. PLoS ONE.

[26] Li W, Wang B, Tan M, Song X, Xie S, Wang C (2022). Analysis of sputum microbial metagenome in COPD based on exacerbation frequency and lung function: a case control study. Respir Res.

[27] Millares L, Pascual S, Montón C (2019). Relationship between the respiratory microbiome and the severity of airflow limitation, history of exacerbations and circulating eosinophils in COPD patients. BMC Pulm Med.

[28] Ying KL, Brasky TM, Freudenheim JL (2022). Saliva and lung Microbiome associations with electronic cigarette use and smoking. Cancer Prev Res (Phila).

[29] Pfeiffer S, Herzmann C, Gaede KI, Kovacevic D, Krauss-Etschmann S, Schloter M (2022). Different responses of the oral, nasal and lung microbiomes to cigarette smoke. Thorax.

[30] Pragman A, Issacson R, Wendt C, Reilly C (2015). A method for determining Taxonomical contributions to Group differences in Microbiomic Investigations. J Comput Biol.

[31] Sundh J, Tanash H, Arian R (2021). Advanced Dental Cleaning is Associated with reduced risk of COPD exacerbations - A Randomized Controlled Trial. Int J Chron Obstruct Pulmon Dis.

[32] Gaeckle NT, Pragman AA, Pendleton KM, Baldomero AK, Criner GJ (2020). The oral-lung Axis: the impact of oral health on Lung Health. Respir Care.

[33] Cohen LA, Bonito AJ, Eicheldinger C (2011). Behavioral and socioeconomic correlates of dental problem experience and patterns of health care-seeking. J Am Dent Assoc.

[34] Gomes S, Cavadas B, Ferreira JC (2019). Profiling of lung microbiota discloses differences in adenocarcinoma and squamous cell carcinoma. Sci Rep.

[35] Jin C, Lagoudas GK, Zhao C (2019). Commensal microbiota promote Lung Cancer Development via γδ T cells. Cell.

[36] Kim OH, Choi BY, Kim DK (2022). The microbiome of lung cancer tissue and its association with pathological and clinical parameters. Am J Cancer Res.

[37] Keir HR, Contoli M, Chalmers JD (2021). Inhaled corticosteroids and the lung Microbiome in COPD. Biomedicines.

[38] Contoli M, Pauletti A, Rossi MR (2017). Long-term effects of inhaled corticosteroids on sputum bacterial and viral loads in COPD. Eur Respir J.

[39] Leitao Filho FS, Takiguchi H, Akata K (2021). Effects of inhaled Corticosteroid/Long-Acting β_2_-Agonist combination on the Airway Microbiome of patients with Chronic Obstructive Pulmonary Disease: a Randomized Controlled Clinical Trial (DISARM). Am J Respir Crit Care Med.

