# Supplementary material for: Tobacco use, self-reported professional dental cleaning habits, and lung adenocarcinoma diagnosis are associated with bronchial and lung microbiome alpha diversity
Source: Respir Res. 2024 Mar 18;25:130. doi: 10.1186/s12931-024-02750-0 (PMC10949571; doi:10.1186/s12931-024-02750-0)
Supplement: Supplementary file 1 — Supplementary Material 1 [file 12931_2024_2750_MOESM1_ESM.docx]

**SUPPLEMENTARY INFORMATION.**

**Supplementary Methods**

**Study design, recruitment, and sample collection procedures.** Our prospective observational study of the upper and lower airway microbiome among individuals undergoing lung resection was undertaken to assess the airway microbiome in a manner that minimizes the upper airway contamination of low biomass lung and bronchial samples. Patients undergoing lung lobectomy at the Minneapolis VA Medical Center (MVAMC) for suspected or confirmed lung cancer who met inclusion/exclusion criteria were approached for consent. Subjects must have been age 40 or older. Subjects were excluded if they had asthma, an endobronchial lesion or lobar atelectasis, or had used antibiotics or systemic corticosteroids in the prior 1 months. Potentially eligible subjects were approached the day prior to surgery. Following documentation of informed consent, subjects provided their medical history, medication use history, and were asked to self-report their tobacco use and professional dental care histories. Subjects were asked to fast for 2 hours (including no toothbrushing) and then to provide oral wash 1 sample by swishing 10 ml of sterile water in their mouth for 30 seconds and spitting it into sterile, DNA-free tubes. Subjects underwent sputum induction with 3% saline for up to 20 minutes and sputum samples were collected in sterile, DNA-free tubes. A negative control sample, consisting of unused sterile water placed in a sample tube, was also collected. The morning of surgery (after an overnight fast), oral wash 2 was collected as for oral wash 1. In the operating room, the resected lung was handed off the operative table in a sterile basin. Sterile instruments were used to cut open the staple line sewing the bronchus shut. Tissue sampling was performed without placing the tissue in media. A sterile nylon-flocked swab (Copan Diagnostics, Murrieta, CA) was used to swab the bronchial airway for 15 seconds, then the swab was placed in a sterile, DNA-free container. Lung parenchymal samples were obtained by cutting open the distal lung tissue and vigorously swabbing the alveolar air spaces for 30 seconds. Care was taken to avoid the suspected malignancy (if it was still present in the lobectomy sample) and to avoid swabbing the pleural surfaces. This process was repeated twice more at different lung parenchymal sites for the other two lung samples. Then nasopharyngeal samples were obtained using by swabbing the nasopharynx for 15 seconds. A negative control swab, consisting of an unused swab opened in the operating room and placed in a collection tube was also obtained. All samples were placed on ice after collection, then transported to the laboratory and frozen at -70 °C until DNA extraction. Pulmonary function test results and pathology results were obtained via chart review. The protocol was approved by the Minneapolis VA IRB (#4348-B).

**Sample processing.** Prior to DNA extraction, care was taken to avoid batch effects by ensuring that subject replicates were not extracted in the same batch. All samples were extracted using the MO BIO PowerSoil DNA Isolation Kit (QIAGEN, Germantown, MD) with modifications as noted below. We monitored for individual kit effects by recording the kit used to extract each sample. Samples obtained on swabs (nasopharynx, bronchus, and lung) were thawed and transferred to a PowerBead tube followed by addition of PowerSoil solution C1 and incubation at 70 °C for 15 minutes. Oral wash and sputum samples were massed for subsequent normalization of 16S copy numbers to sample mass. Oral wash samples were centrifuged at 13,000g for 15 minutes to pellet the sample, then resuspended in PowerBead tube buffer solution and transferred back to the tube. Sputum samples were incubated at 37 °C with an equal volume of Sputolysin (Millipore-Sigma, Darmstadt, Germany) for 15 minutes, then pelleted at 13,000g for 15 minutes and resuspended in PowerBead tube solution for transfer into the tube. Once placed in the PowerBead tubes, the samples were extracted following the manufacturer’s instructions.

**16S rRNA gene quantification and sequencing.** Extracted DNA from each sample was submitted to the University of Minnesota Genomics Center for 16S rRNA gene quantification and 16S rRNA gene V4 MiSeq sequencing. 16S rRNA gene quantification was accomplished with droplet digital PCR (ddPCR) on the Bio-Rad QX200 instrument using a standard curve created with serial dilutions of *E. coli* template DNA tested in triplicate. PCR was accomplished using the 2X EvaGreen SuperMix (Bio-Rad) and 16S rRNA V4 primers (GTGCCAGCMGCCGCGGTAA, GGACTACHVGGGTWTCTAAT). ddPCR reactions were performed as follows (ramp rate of 2 °C/second): 95 °C for 5 minutes; followed by 45 cycles of 95 °C for 30 seconds/60 °C for 90 seconds; then 4 °C for 5 minutes followed by 90 °C for 5 minutes. All samples, including negative control or subject samples with very low 16S rRNA copy numbers, were sequenced as below. Libraries were prepared for 16S rRNA V4 marker gene sequencing on the MiSeq instrument using dual-indexing as described previously,(1) but modified to use ultra-clean production (UCP) reagents and a touchdown PCR protocol to optimize sequencing of low biomass samples. The touchdown PCR protocol consisted of 98 °C for 30 seconds; then 17 cycles of 98 °C for 20 sec/60 °C for 20 sec/72 °C 1 minute (decrease 0.3 °C per cycle); then 23 cycles of 98 °C for 20 sec./55 °C for 20 sec./72 °C for 1 min.; then 72 °C for 5 min.

**16S rRNA sequence processing**. Sequences were trimmed for sequencing artifacts and primers with cutadapt (https://cutadapt.readthedocs.io/en/stable/#). Further quality trimming, error correction, chimeric read removal, and ASV table creation were done with DADA2.(2) CLR transformation was used prior to α- and β-diversity calculations. (3) Taxonomic identification was done with dada2 using RDP Taxonomy 18.(4) Additional ASV table processing was done using R as described here: https://github.com/trevorjgould/MarkerGeneAnalysis. The R package decontam was used to understand potential contaminant taxa (Figure S1 (5). The prevalence method with default threshold of 0.1 was chosen due to the low biomass samples in our dataset (i.e., the prevalence method is most appropriate for low biomass lung and bronchus samples). Additionally, samples processed from swabs were analyzed independently of samples processed from liquids because different extraction techniques may lead to different contaminants (i.e., samples obtained via swab [nasopharynx, bronchus, lung, and negative control swabs] were analyzed independently of the samples obtained as liquids [oral wash, sputum, and negative control liquid samples]). Among the liquid samples and controls, 14 sequences were identified as contaminants. 7 of them were not classified at the phylum level (indicating that these were likely sequencing errors), and only 2 of the 14 sequences were also identified in the swab dataset. Among the liquid samples, the identified contaminants were in the genera *Staphylococcus, Escherichia/Shigella, Lawsonella,* or *Cornyebacterium*. Among the samples and controls obtained from swabs, 14 sequences were identified. Only 2 were also identified in the liquid dataset. Of the 14 sequences, 4 were not classified at the phylum level. The two sequences identified in both datasets were *Pseudomonas* and “not classified” at the phylum level. The remaining 8 sequences were in the genera *Veillonella, Neisseria, Mycoplasmoides, Corynebacterium,* or *Prevotella*. These genera are expected members of the airway microbiome. Therefore, we chose to exclude only those sequences not classified at the phylum level, but did not exclude any others as there was not consistent evidence that they were contaminants and the identified sequences were expected members of the upper or lower airway microbiome. Removal of the unclassified sequences resulted in removal of 10 of the 26 potential contaminants identified by decontam. Subsequent analyses, including all the analyses described in the manuscript and below, were conducted after excluding the unclassified sequences from all samples and controls (Figure S2).

α-diversity metrics were obtained using OTUtable (Chao1) and Vegan (Shannon diversity index, Simpson diversity index). β-diversity was assessed using the Bray-Curtis matrix with CLR transformation followed by principal component analysis as outlined here: https://github.com/trevorjgould/MarkerGeneAnalysis/blob/master/R/diversity.R.

**Statistical analyses.** All analyses were conducted in R version 3.6.0. Linear regression (LR) was used for analyses with independent data points. Analyses incorporating repeated measures from the same subject employed generalized estimating equation (GEE) with independence correlation structure. Where relevant, p-values were adjusted for multiple hypothesis testing as below.

*Baseline characteristics.* Frequencies and percentages are presented unless specified otherwise. A two-sample t test was conducted for all continuous variables and a Fisher exact test was conducted for all categorical variables.

*16S rRNA copy number analyses.* All undetectable samples were assigned a raw copy number of 1, and detectable samples with <10 copies (the limit of quantification) were assigned a raw copy number of 5. For oral wash and sputum samples, raw 16S copy numbers were normalized to sample mass and then converted to log_10_ scale for all analyses. For samples obtained on swabs (nasopharynx, lung, and bronchus) raw 16S copy numbers converted to log_10_ scale were used. Summary statistics are provided (Table S1). Comparisons were made using a GEE model with *post-hoc* comparisons. P values were adjusted with the single-step method.

*α-diversity analyses.* Simpson diversity index, Shannon diversity index, and Chao1 diversity index were reported for each sample (Figure S3). Within-subject associations by anatomic site were determined with GEE analysis (Table S2). Associations with clinical factors were assessed using a GEE model (when diversity was compared across anatomic sites or at an anatomic site with more than one sample collection [oral wash, lung]) or LR model (when diversity was compared at an anatomic site with one sample collection).

*β-diversity analyses*. Bray-Curtis dissimilarity and principal coordinate analysis (PCoA) was used to illustrate microbial composition and between-sample similarity. Initial PCoA analyses were performed with negative control samples included. Separate PCoA plots were created for the samples and negative controls obtained in liquid form (oral washes, sputa, and sterile water negative controls) and those obtained from nylon-flocked swabs (nasopharynx, bronchus, lung, and unused control swabs; Figure S2). Subsequently, all subject samples were combined in one dataset and the negative control samples were removed before the Bray-Curtis dissimilarity matrix was re-calculated. PERMANOVA analyses restricted to individual sample sites were performed using the Bray-Curtis dissimilarity matrix recalculated for each site (Figure S6).

*Taxonomic composition*. Samples were split by site (oral wash, bronchus, lung, nasopharynx, and sputum). The datasets were filtered to remove genera present in <10% of samples at each anatomic site independently. For sites with multiple samples (oral wash, lung), we eliminated genera present in <10% of samples and then used GEE models with log (reads+1) as the response. For the other sites with only one sample per site (nasopharynx, bronchus, sputum) we removed genera present in <10% of samples, and then used linear regression models with log (reads+1) as the response. Clinical characteristics were used as explanatory variables at each anatomic site and characteristic independently. Dental cleaning was analyzed as a binary variable (within the last 6 months vs. more than 6 months prior). Adenocarcinoma was analyzed as a binary variable (has adenocarcinoma vs no-adenocarcinoma). P-values were adjusted using the Holm method.

**Supplementary Results:**

**Study Samples.** The 44 subjects were each asked to provide 8 samples (2 oral washes, 1 sputum, 1 nasopharyngeal swab, 1 bronchial swab, and 3 lung parenchymal swabs) from 5 different sites (oral, sputum, nasopharyngeal, bronchial, and lung parenchyma). Eight subjects did not provide sputum samples either because they declined sputum induction (3), the investigator felt sputum induction carried undue risk (1), or the subject was unable to produce a sample during the induction procedure (4). Four subjects (3 with COPD, 1 without COPD) did not provide lung and bronchial samples. In 3 of these subjects the samples were not obtained because they did not undergo lobectomy for clinical reasons. In 1 subject the samples were not obtained because the clinical staff placed the resected lobe in formalin before the study team could obtain study samples. Therefore, 40 of 44 subjects provided lung and bronchial samples.

**Identification of potential contaminants.** As described in the above supplemental methods, sequences that were not classified at the phylum level were removed from the dataset prior to any additional analyses. Then, negative control sample composition was compared to study sample composition utilizing principal coordinate analysis (PCoA) and stacked bar charts. Two sets of PCoA plots and bar charts were constructed, with samples and controls chosen based on sample biomass and sample extraction technique. Samples obtained as liquids (oral wash, sputum) were plotted with liquid extraction controls while samples obtained using swabs (nasopharynx, bronchus, lung) were plotted with swab extraction controls (Figure S2). As expected, among the higher-biomass liquid samples the negative control samples separated from subject samples. Among the lower biomass samples obtained via swab, negative control samples clustered separately from nasopharyngeal samples, with some similarities to low biomass lung and bronchial samples.

**Supplementary Figures.**

**Figure S1. Decontam output.**

**
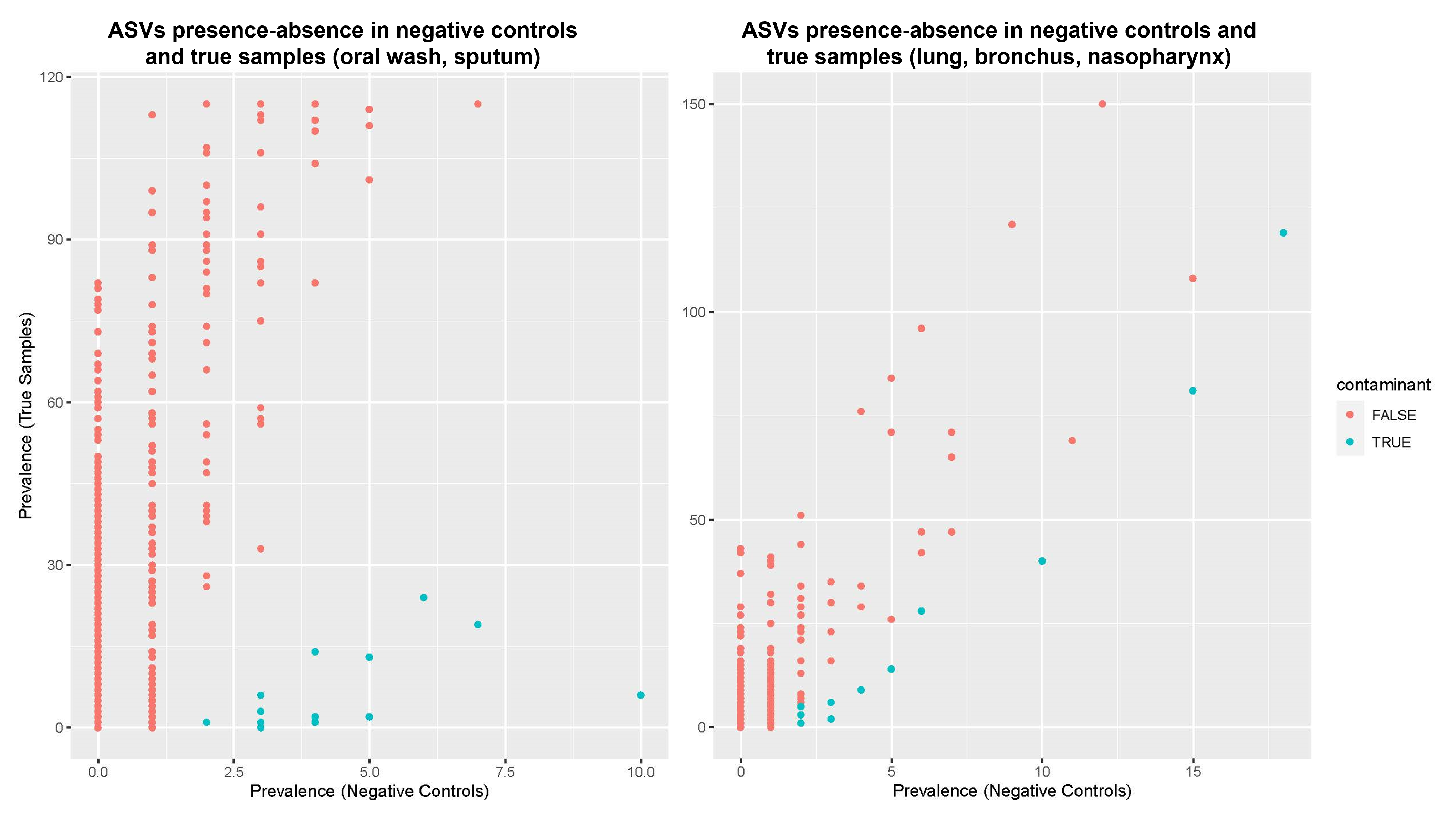
**

**Figure S2. Principal coordinate analysis (PCoA) and taxonomic comparison of subject samples and negative controls.**

**
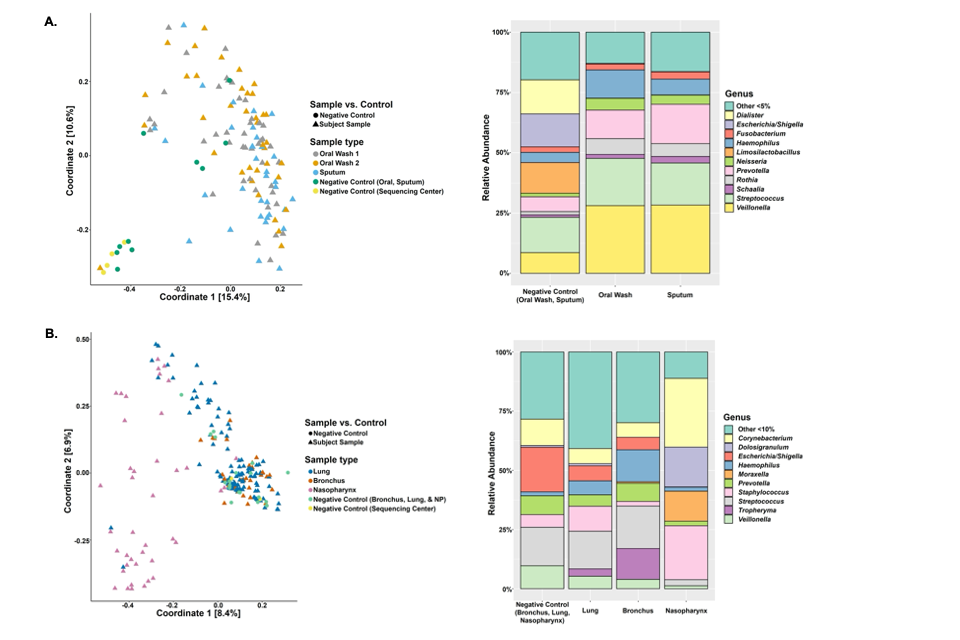
**

**Figure S3. Oral wash and sputum samples have greater α-diversity than lung, bronchus, and nasopharyngeal samples.**

**
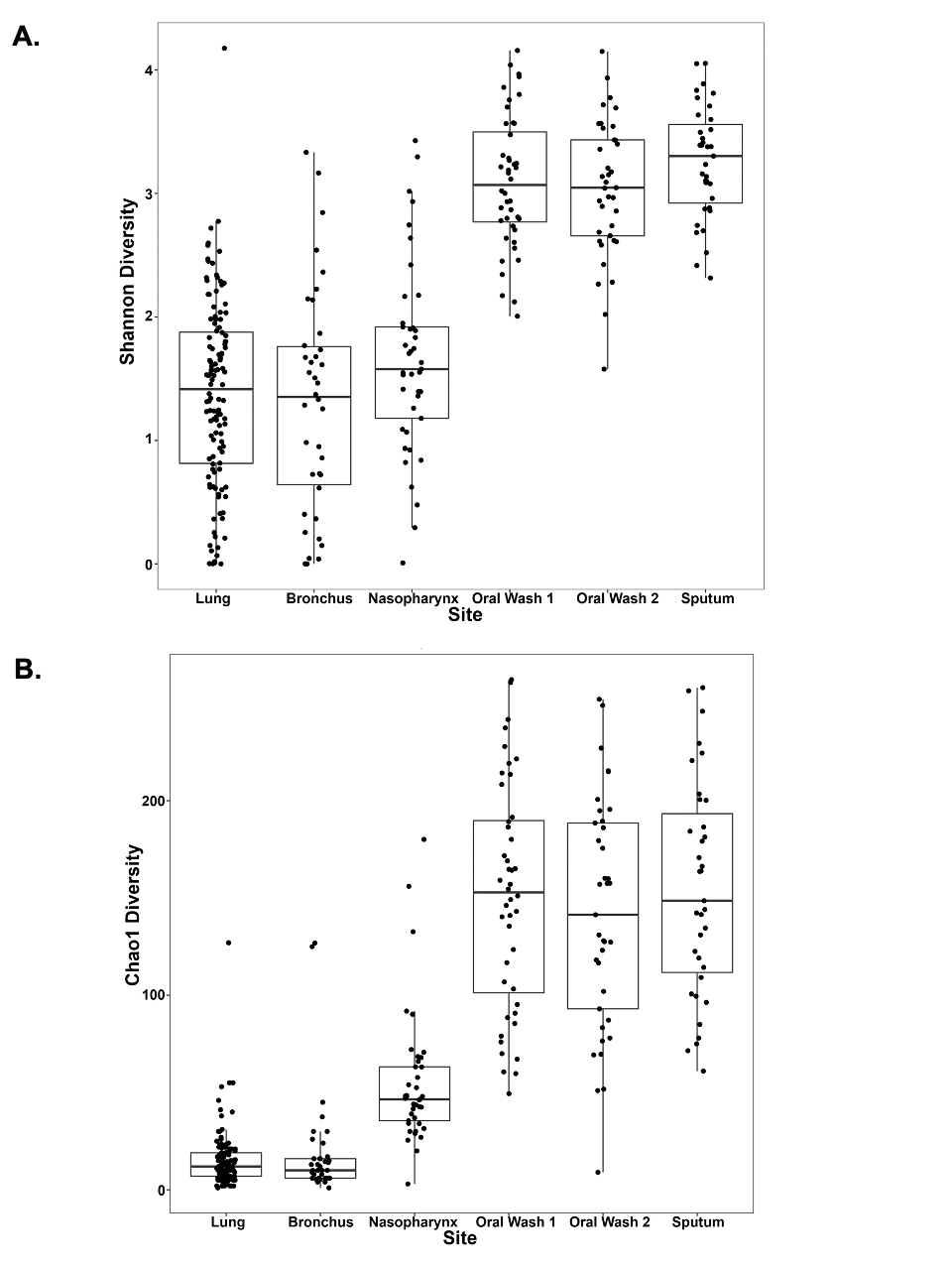
**

**Figure S4. Oral wash α-diversity of dentate vs. edentulous subjects.**

**
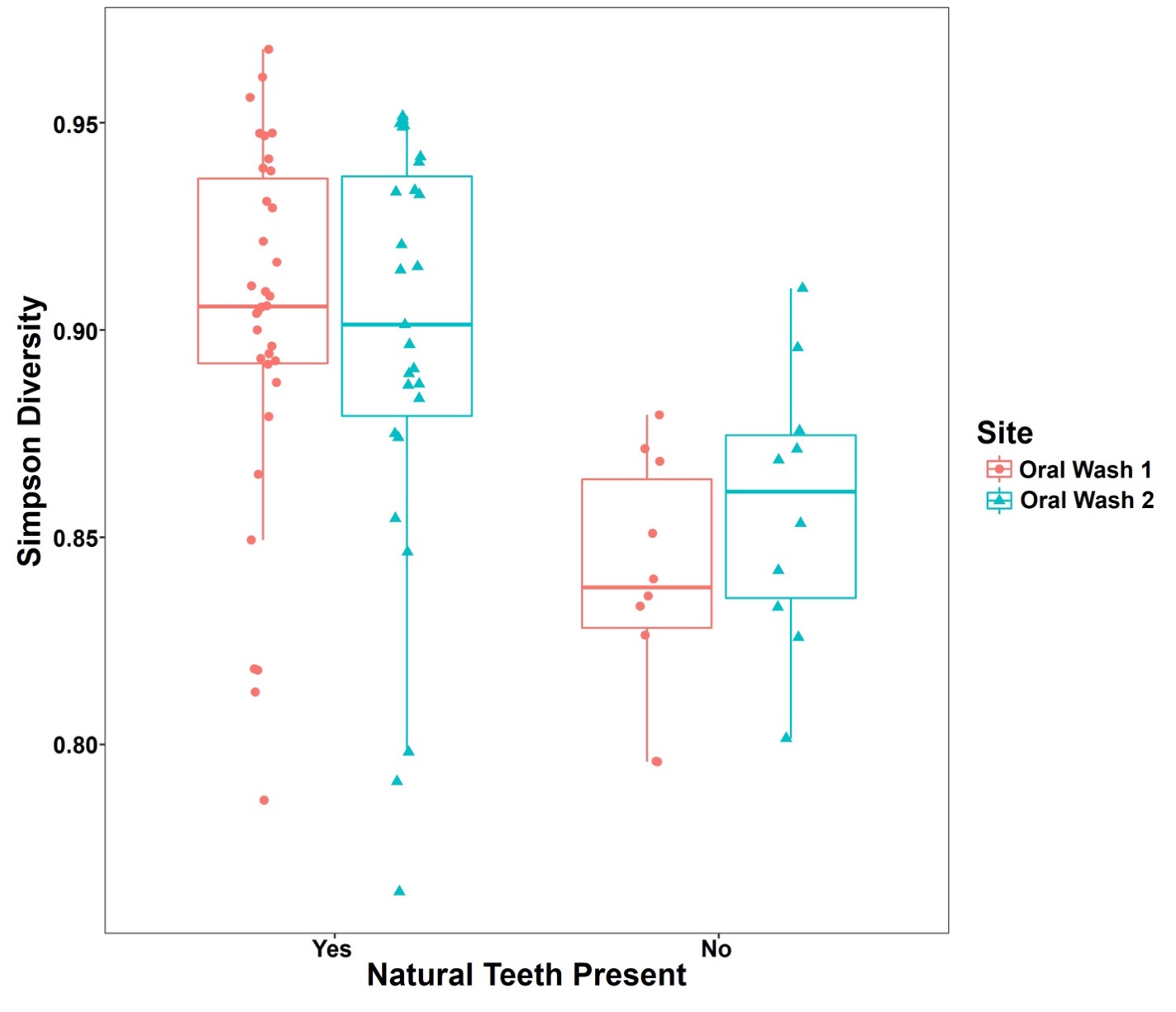
**

**Figure S5. Weighted UniFrac illustrates sample clustering by site.
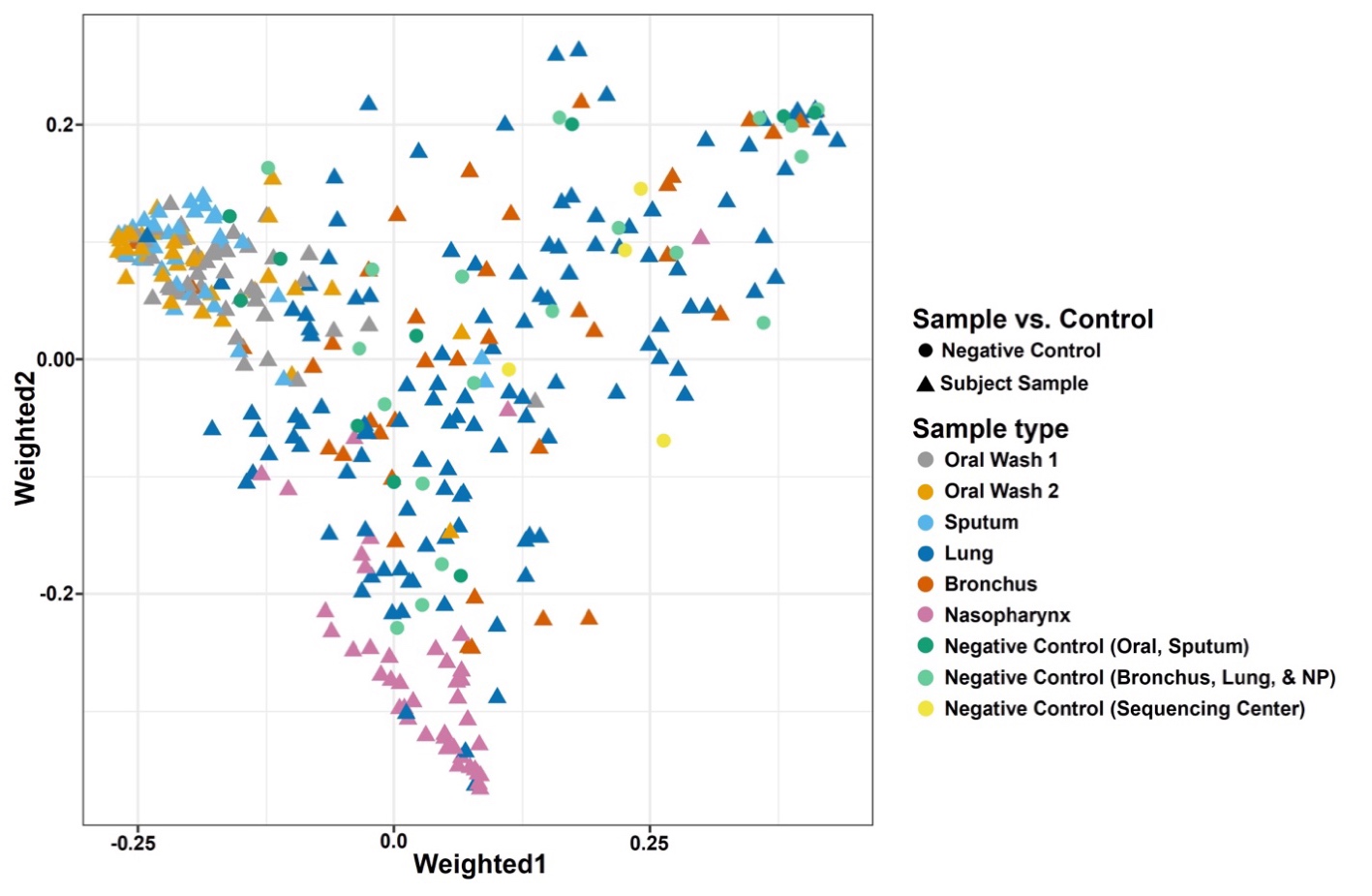
**

**Figure S6. Principal coordinate analysis (PCoA) with Bray-Curtis dissimilarity revealed potential associations between clinical characteristics and microbiome composition.**

**
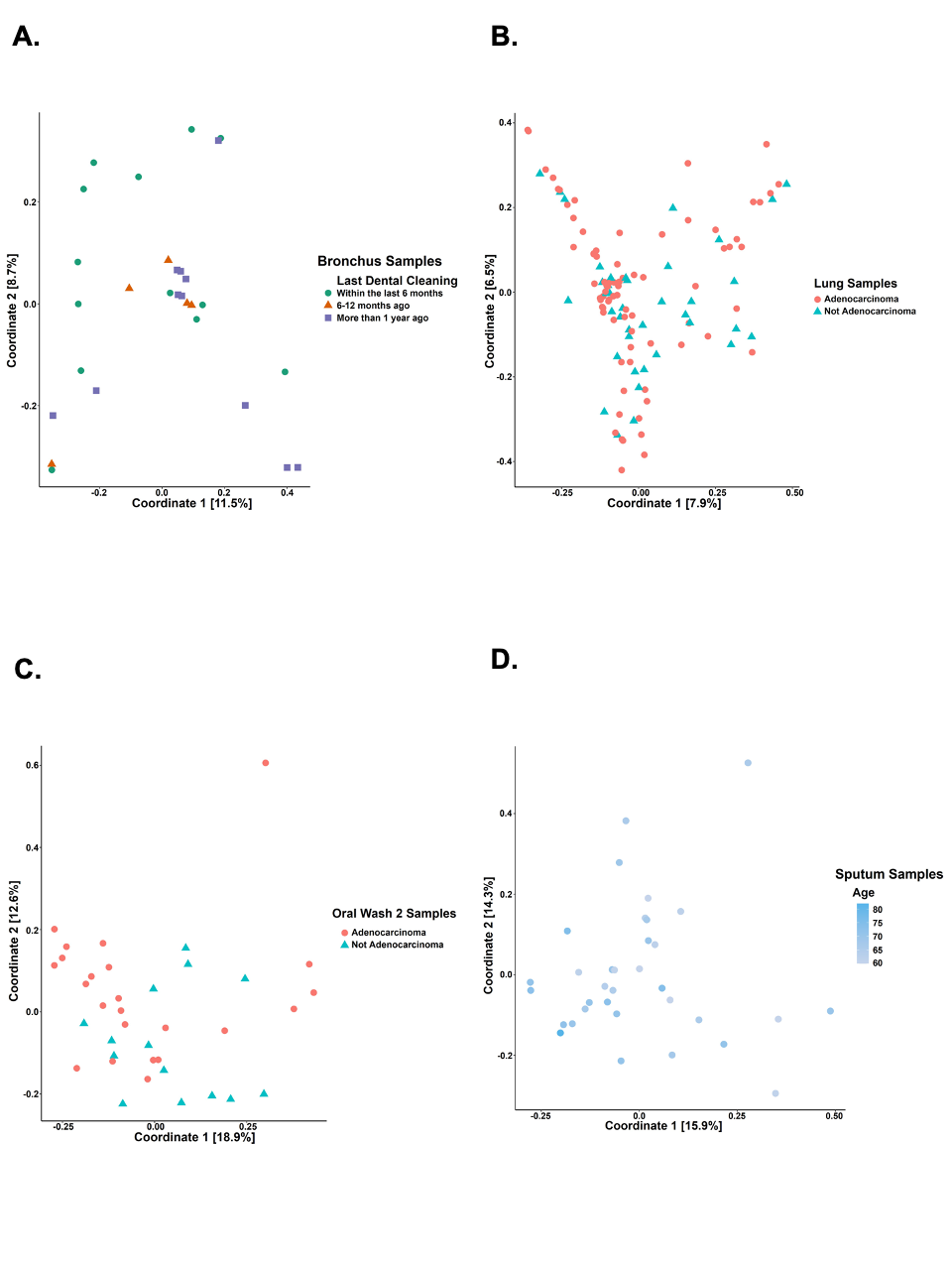
**

**Supplementary Figure Legends.**

**Figure S1. Decontam output.** The R package decontam was used to understand potential contaminant taxa as described above. The prevalence method with default threshold of 0.1 was chosen due to the low biomass samples in our dataset. Additionally, samples processed from swabs were analyzed independently of samples processed from liquids because different extraction techniques may have led to different contaminants.

**Figure S2. Principal coordinate analysis (PCoA) and taxonomic comparison of subject samples and negative controls.** Following removal of contaminant taxa, the β-diversity of all subject samples and negative control samples was illustrated with PCoA. PCoA differentiated negative control samples and subject samples by shape, with sample type illustrated by color. Taxonomic composition of subject samples and negative controls was illustrated by stacked bar chart. Stacked bar charts were created by merging all sequences from each anatomic site or relevant negative control; multiple ASVs from the same genus were combined. **A**. Oral wash, sputum, and relevant negative control sample β-diversity was calculated using Bray-Curtis dissimilarity and illustrated with PCoA. Coordinate 1 represents 15.4% of the variance and coordinate 2 represents 10.6% of the variance. Stacked bar charts were created by merging all sequences from each site; multiple ASVs from each genus were combined. Stacked bar charts represent the relative abundance of taxa present at each site. Taxa present at <5% relative abundance were combined as “other” taxa. **B**. Lung, bronchus, nasopharynx, and relevant negative control sample β-diversity was calculated using Bray-Curtis dissimilarity and illustrated with PCoA. Coordinate 1 represents 8.4% of variance while coordinate 2 represents 6.9% of variance. Stacked bar charts represent the relative abundance of taxa present at each site. Taxa present at <10% relative abundance were combined as “other” taxa.

**Figure S3. Oral wash and sputum samples have greater α-diversity than lung, bronchus, and nasopharyngeal samples.** Horizontal bars represent the median value for each sample while the top and bottom of the boxes represent the 75^th^ and 25^th^ percentile values, respectively. **A.** Shannon diversity was determined for each sample and illustrated by sample site. Oral wash and sputum samples are significantly more diverse than lung, bronchus, and nasopharyngeal samples (GEE, p<0.0001). **B.** Chao1 diversity was determined for each sample and illustrated by sample site. Oral wash, sputum, and nasopharyngeal samples have greater richness than lung and bronchus samples (GEE, p<0.0001).

**Figure S4. Oral wash α-diversity of edentulous vs. dentate subjects.** Horizontal bars represent the median value for each sample while the top and bottom of the boxes represent the 75^th^ and 25^th^ percentile values, respectively. Oral wash 1 and oral wash 2 samples are represented by color for both edentulous (n=10) and dentate (n=34) subjects. Edentulous subjects’ oral wash samples were not included in subsequent analyses of professional dental care habits and the lower airway microbiome.

**Figure S5. Weighted UniFrac illustrates sample clustering by site.** All subject samples and negative control samples were assessed by weighted UniFrac. Shape is used to distinguish samples from negative controls while color is used to distinguish among anatomic sites. Lung and bronchus samples are similar to each other, and cluster separately from the nasopharyngeal samples and the oral wash and sputum samples.

**Figure S6. Principal coordinate analysis (PCoA) with Bray-Curtis dissimilarity revealed potential associations between clinical characteristics and microbiome composition.** Several clinical characteristics resulted in p-values between 0.05 and 0.10, which did not reach statistical significance. These include potential associations between **A**. dental cleaning and bronchial microbiome β-diversity (p=0.09, R^2^=0.046), **B**. the diagnosis of lung adenocarcinoma (vs. other diagnoses) and lung microbiome composition (p=0.054, R^2^=0.011) or **C**. oral wash 2 composition (p=0.096, R^2^=0.040), and **D**. age and the sputum microbiome composition (p=0.057, R^2^=0.047).

**Supplementary Tables.**

**Table S1. Summary Statistics of 16S Copy Numbers for Samples and Negative Controls.**

| Sample type | Mean | Median | IQR |
| --- | --- | --- | --- |
| Negative control (bronchus, lung, nasopharynx) | 8.53 | 2.79 | 8.77 |
| Negative control (oral wash, sputum) | 58.89 | 1.84 | 3.53 |
| Bronchus | 437.94 | 239.31 | 537.01 |
| Lung | 935.02 | 89.53 | 317.13 |
| Nasopharynx | 35,668.79 | 19,641.57 | 23,293.94 |
| Oral Wash 1 | 4,016,470 | 1,680,760 | 3,729,177 |
| Oral Wash 2 | 6,966,928 | 4,101,593 | 5,354,818 |
| Sputum | 2,991,780 | 1,038,179 | 2,177,690 |

**Table S2. Within-Subject Simpson Diversity Associations by Anatomic Site^a^.**

| Anatomic Site | Coefficient | SE | 95% CI | P value |
| --- | --- | --- | --- | --- |
| Lung | 0.0584 | 0.0535 | -0.046, 0.16 | 0.275 |
| Nasopharynx | 0.1076 | 0.0601 | -0.010, 0.23 | 0.074 |
| Oral Wash 1 | 0.3482 | 0.0489 | 0.25, 0.44 | <0.0001 |
| Oral Wash 2 | 0.3466 | 0.0480 | 0.25, 0.44 | <0.0001 |
| Sputum | 0.3635 | 0.0475 | 0.27, 0.46 | <0.0001 |

^a^Reference site is bronchus

**Table S3. PERMANOVA Analyses Based on Weighted UniFrac^a^.**

| **Characteristic** | **Lung** | | **Bronchus** | | **Sputum** | | **Oral Wash 2** | | **Nasopharynx** | |
| --- | --- | --- | --- | --- | --- | --- | --- | --- | --- | --- |
|  | **R^2^** | **p-value** | **R^2^** | **p-value** | **R^2^** | **p-value** | **R^2^** | **p-value** | **R^2^** | **p-value** |
| Last professional dental cleaning | **0.0217** | **0.034** | 0.0625 | 0.085 |  |  |  |  |  |  |
| Adenocarcinoma | **0.0227** | **0.004** |  |  |  |  | 0.0489 | 0.104 |  |  |
| Age |  |  |  |  | **0.0743** | **0.016** |  |  | 0.0515 | 0.081 |

^a^Only sites and characteristics with p-values ≤0.10 are reported here.

**REFERENCES**

1. Gohl DM, Vangay P, Garbe J et al. Systematic improvement of amplicon marker gene methods for increased accuracy in microbiome studies. Nature Biotechnology. 2016;34:942-949.

2. Callahan BJ, McMurdie PJ, Rosen MJ, Han AW, Johnson AJ, Holmes SP. DADA2: High-resolution sample inference from Illumina amplicon data. Nat Methods. 2016;13:581-583.

3. Gloor GB, Macklaim JM, Pawlowsky-Glahn V, Egozcue JJ. Microbiome Datasets Are Compositional: And This Is Not Optional. Front Microbiol. 2017;8:2224.

4. Wang Q, Garrity GM, Tiedje JM, Cole JR. Naive Bayesian classifier for rapid assignment of rRNA sequences into the new bacterial taxonomy. Appl Environ Microbiol. 2007;73:5261-5267.

5. Davis NM, Proctor DM, Holmes SP, Relman DA, Callahan BJ. Simple statistical identification and removal of contaminant sequences in marker-gene and metagenomics data. Microbiome. 2018;6:226.
